# Polycomb Protein OsFIE2 Affects Plant Height and Grain Yield in Rice

**DOI:** 10.1371/journal.pone.0164748

**Published:** 2016-10-20

**Authors:** Xianbo Liu, Xiangjin Wei, Zhonghua Sheng, Guiai Jiao, Shaoqing Tang, Ju Luo, Peisong Hu

**Affiliations:** State Key Laboratory of Rice Biology, Key Laboratory of Rice Biology and Breeding of Ministry of Agriculture, China National Rice Research Institute, Hangzhou, 310006, China; Institute of Botany Chinese Academy of Sciences, CHINA

## Abstract

Polycomb group (PcG) proteins have been shown to affect growth and development in plants. To further elucidate their role in these processes in rice, we isolated and characterized a rice mutant which exhibits dwarfism, reduced seed setting rate, defective floral organ, and small grains. Map-based cloning revealed that abnormal phenotypes were attributed to a mutation of the Fertilization Independent Endosperm 2 (OsFIE2) protein, which belongs to the PcG protein family. So we named the mutant as *osfie2-1*. Histological analysis revealed that the number of longitudinal cells in the internodes decreased in *osfie2-1*, and that lateral cell layer of the internodes was markedly thinner than wild-type. In addition, compared to wild-type, the number of large and small vascular bundles decreased in *osfie2-1*, as well as cell number and cell size in spikelet hulls. *OsFIE2 is* expressed in most tissues and the coded protein localizes in both nucleus and cytoplasm. Yeast two-hybrid and bimolecular fluorescence complementation assays demonstrated that OsFIE2 interacts with OsiEZ1 which encodes an enhancer of zeste protein previously identified as a histone methylation enzyme. RNA sequencing-based transcriptome profiling and qRT-PCR analysis revealed that some homeotic genes and genes involved in endosperm starch synthesis, cell division/expansion and hormone synthesis and signaling are differentially expressed between *osfie2-1* and wild-type. In addition, the contents of IAA, GA_3_, ABA, JA and SA in *osfie2-1* are significantly different from those in wild-type. Taken together, these results indicate that *OsFIE2* plays an important role in the regulation of plant height and grain yield in rice.

## Introduction

Rice (Oryza sativa) has been a hot spot in plant science research because it is considered a main staple food for more than half of the world’s population. Its yield is mainly determined by grain weight, spike number and number of grains per panicle, but also can be affected by plant height and flowering time [1]. To date, numerous genes associated with spike number and number of grains per panicle, such as *GN1A*, *TAW1*, *DEP1*, *OsSPL14*, *Ghd7*, *DTH8/Ghd8* have been isolated and characterized [1–4]. On the other hand, grain weight is determined by grain size and ratio of grain filling. Recently, many grain size quantitative trait loci (QTLs) have been identified and characterized. *GS3* and *GS5* regulate grain size [5, 6], *GW2*, *GW5*/*qSW5* and *GW8* control grain width [7–9], *qGL3*/*qGL3*.*1*, GL7 and *GLW7* affect grain length [10–13]. These genes are involved mainly in signaling pathways mediated by proteasome degradation, phytohormones and G proteins to regulate cell proliferation and cell elongation [14]. Others such as *GIF1* and *TGW6* were considered to regulate the degree of grain filling [15, 16]. Because of the complexity of the genetic mechanism underlying grain weight, regulation pathways have not yet been fully clarify consequently the identification and characterization of other genes related to grain weight will be useful to generating high yield cultivars.

Plant height is also a crucial trait for grain yield in modern agriculture [17]. Rice ‘green revolution’ had a positive impact in increasing the yield potential of rice, which has been represented by breeding of dwarf cultivars [18, 19]. To date, a large number of dwarf mutants have been identified and characterized in rice, with most being related to the biosynthesis and responsiveness to phytohormones [20]. Dwarf mutants such as *sd1*, *d18*, *d35*, *slr1*, *gid1* and *gid2* are affected in the biosynthesis of or responsiveness to gibberellic acids (GAs) [21–26]. The genes *D2*, *D11*, *BRD1*, *BRD2*, *OsDWARF4*, *RAVL1*, *BZR1*, *LIC1*, *BU1*, *TUD1*, *DLT*, *BAK1* and *OsBRI1* are involved in biosynthesis or signaling pathways of brassinolide (BL) [27]. While other rice genes such as *D10*, *D17*/*HTD1*, *D27*, *D3*, *D14*/*HTD2*, *D53* are implicated in the biosynthesis or signaling of strigolactones (SLs), a recently discovered group of plant regulators that control shoot branching [28, 29]. Despite this, exploring for new genetic mechanisms controlling rice plant height is still research focus in rice genetics and genomics.

Polycomp group (PcG) proteins are one of the chromatin regulation factors first reported in *Drosophila melanogaster* [30]. PcG proteins play essential roles in animal and plant life cycles by controlling the expression of important developmental regulators, as well as by regulating cell proliferation [31–34]. PcG proteins are composed of three forms of multiprotein complex, polycomb repressive complex 1 (PRC1), polycomb repressive complex 2 (PRC2) and pleiohomeotic repressive complex (Pho RC) [32, 35, 36]. PRC2 consists of at least four core components: Enhancer of Zeste (E (Z)), Suppressor of Zeste 12 (Su (Z)) 12, Extra Sex combs (ESC) and Nucleosome remodeling factor 55 (Nurf 55). [37]. Both E (Z) and Su (Z) have three homologs in *Arabidopsis thaliana*, while there is only one homolog for ESC and Nurf55, Fertilization Independent Endosperm (FIE) and Multicopy Suppressor of Ira1 (MSI1), respectively. FIE and other three core components form three PRC2-like complexes including: the Fertilization Independent Seed (FIS), Embryonic Flower (EMF) and Vernalization (VRN) complexes [38–45]. The rice genome contains two genes for ESC (*OsFIE1* and *OsFIE2*) [46]. *OsFIE1* is an endosperm-specific gene, involved in H3K27me3-mediated gene repression. It is regulated by DNA methylation and histone H3K9me2 and its ectopic expression causes a dwarf and floral defect [47]. *OsFIE2*, a homolog of *OsFIE1*, regulates seed development and grain filling [33, 46, 48]. The *OsFIE2* RNAi lines shown pleiotropic phenotypes in vegetative and reproductive organ generation, such as dwarf, abnormal enlarge lemma, but the RNAi lines for *OsFIE2* reduces gene expression both in *OsFIE1* and *OsFIE2* [33]. So, *osfie2* mutant is more helpful to understand functions of rice PRC2 protein.

In this study, we isolated a rice OsFIE2 mutant, *osfie2-1*, which exhibited a dwarf phenotype as well as reduced seed setting rate, abnormal floral organs and small grains. We further demonstrate that OsFIE2 protein plays an important role in regulating plant height and grain yield.

## Materials and Methods

### Plant materials and field experiments

The dwarf and small grain mutant *osfie2-1* was obtained from an EMS-induced mutant population of *japonica* rice cv. Zhonghua 11. The F_1_ plants and F_2_ populations derived from the cross between *osfie2-1* and cv. Dular, reciprocal crosses between *osfie2-1* and its wild-type were used for genetic analysis of the mutant gene. The F_2_ generated from the cross between *osfie2-1* and cv. Dular also used for gene fine-mapping. All rice plants were cultivated in paddy fields under natural conditions (China National Rice Research Institute, Hangzhou). A total of 12 plants for each genotype, *osfie2-1* and wild-type, were used to evaluate phenotypic data, which included plant height, grain size (length, width and thickness), 1000-grain weight, seed-setting rate and panicle length.

### Histological analyses

The second internodes of the *osfie2-1* and wild-type were sampled and fixed in formalin-acetic acid-alcohol (FAA) overnight at 4°C and then dehydrated in a graded alcohol series (70%, 80%, 95%, 100%). The samples were embedded in a resin based on Technovit 7100 semi-thin section kit (http://www.emsdiasum.com/), followed by sectioning with an ultramicrotome (Leica, http://www.leica.com/). The 2-μm thick sections were stained with 0.1% toluidine blue and observed using a light microscope. For scanning electron microscopy analysis, spikelet hulls were fixed in a 2.5% glutaraldehyde solution for more than 2 h and then dehydrated in a graded alcohol series. The samples were critical-point-dried, mounted, gold-sputter-coated, and then observed and photographed under a KYKY-EM3200 scanning electron microscope.

### Positional cloning of *osfie2-1*

A 32-plants F_2_ progeny which displayed a similar *osfie2-1* mutant phenotype derived from the crosses between *osfie2-1* and Dular and were used for rough linkage analysis, and subsequently a F_2_ progeny of 603 individuals was used for fine mapping. The positional cloning strategy was described by Zhang et al. (1994)[49]. PCR conditions for amplification of genomic DNA were as follows: 10μl reactions contained 25 ng of template DNA, 1.0 μl 10 X PCR buffer, 0.1 mM dNTPs, 0.1 μM of primer pairs and 0.1 U Taq DNA polymerase. The amplification protocol included an initial denaturation at 95°C for 3 min, followed by 35 cycles at 95°C for 30 s, 30 s annealing at 55°C, and 72°C for 30 s, and a final extension step at 72°C for 5 min. PCR products were separated on a 6% polyacrylamide gel, and silver-stained for visualization. The molecular markers including SSR and Indel markers used for mapping of *osfie2-1* are listed in S2 Table.

### Vector construction and rice transformation

A 1.1kb cDNA corresponding to the full-length ORF of *OsFIE2* was PCR-amplified using two primers (p1300-OsFIE2F/R) and then inserted into the binary vector pCUbi1390 (Ubi promoter inserted into the pCAMBIA1390 vector). The resulting vector was introduced into the rice *osfie2-1* mutant background via Agrobacterium-mediated transformation. The primers for vector constructions are described in S4 Table.

### Yeast two-hybrid Assay

Coding regions of the *OsFIE2* gene was cloned into the ‘bait’ pGBKT9 vector while *OsiEZ1* and *OsCLF* were cloned into the ‘prey’ pGADT7 vector. Reciprocal genetic constructs were also generated (*OsiEZ1* and *OsCLF* into pGBKT9 and *OsFIE2* into pGADT7). The yeast two-hybrid assay was performed following the manufacturer’s instructions (http://www.clontech.com/). The co-transformations with prey and bait were examined on control media, and the interactions between bait and prey were performed on selective media. The primers for vector constructions are described in S4 Table.

### Bimolecular fluorescence complementation

Full-length *OsFIE2* and *OsiEZ1* were amplified by PCR, and inserted into the binary vectors pSPYNE and pSPYCE to obtain the OsFIE2-CY and OsiEZ1-NY constructions. Both OsFIE2-CY and OsiEZ1-NY were co-expressed in rice protoplasts. The yellow fluorescent signals were observed and photographed under laser confocal scanning microscope. The wavelengths for eYFP detection were 488 nm (excitation) and 527 nm (emission). The primers for vector constructions are described in S4 Table.

### RNA extraction and qRT-PCR analysis

Total RNA from seedling stage and heading stage of various tissues was isolated using the Trizol method according to manufacturer's instructions (Invitrogen, www.invitrogen.com). 2μg of DNaseI-treated RNA was used for cDNA synthesis with First Strand cDNA Synthesis kit (Toyobo, http://www.bio-toyobo.cn/). Real time RT-PCR was performed using Thunderbird SYBR qPCR Mix (Toyobo, http://www.bio-toyobo.cn/) and LightCycler 480 Real-time PCR System (Roche, http://www.roche-applied-science.com/). The rice *Ubiquitin* gene (GenBank accession AF184280) was used as the internal control. Oligonucleotide primers are listed in S5 Table.

### RNA-sequencing analysis

Total RNA was extracted using the Trizol reagent (Invitrogen) following the manufacturer's procedure. RNA quantity and purity were analyzed with the Bioanalyzer 2100 and RNA 6000 Nano LabChip Kit (Agilent, CA, USA) with RIN number >7.0. 10 ug of total RNA extracted from *osfie2-1* and wild-type were used to isolate Poly-(A) mRNA using poly-T oligo-attached magnetic beads (Thermo-Fisher). Following purification, the mRNA was cleaved into small pieces using divalent cations and under elevated temperature and used to construct a final cDNA library according to the protocol for the Illumina RNA ligation method (Illumina, San Diego, USA). Briefly, purified RNA with the RNeasy MinElute Kit (Qiagen) was ligated with a pre-adenylated 3' adapter which enables the subsequent ligation of a 5' adapter. Based on the adapter sequence, a reverse transcription reaction followed by PCR was used to create the cDNA constructs. The average size of the paired-end libraries was 300 ± 50 bp. Single end sequencing was performed on an Illumina Hiseq2500 instrument at LC Sciences (Hangzhou, China) following the vendor's recommended protocol. Significant differentially expressed genes were identified considering a p value ≤ 0.05 and a log2 fold-change (log2_FC) ≥ 1. The functional information of these genes were carried out by using Gene Ontology (GO) analysis tool at http://www.geneontology.org/.

### Hormone content analysis

Contents of IAA, GA3, ABA, JA and SA contents were measured in leaves of wild-type and *osfie2-1* at flowering stage. Each sample was composed of at least three plants, and each measurement was repeated three times. The extractions and determination of plant hormones were performed using HPLC-MS as described by Trapp et al (2014) [50].

## Results

### The *osfie2-1* mutant exhibited dwarf phenotype, small grains and floret defects

**Fig 1:**
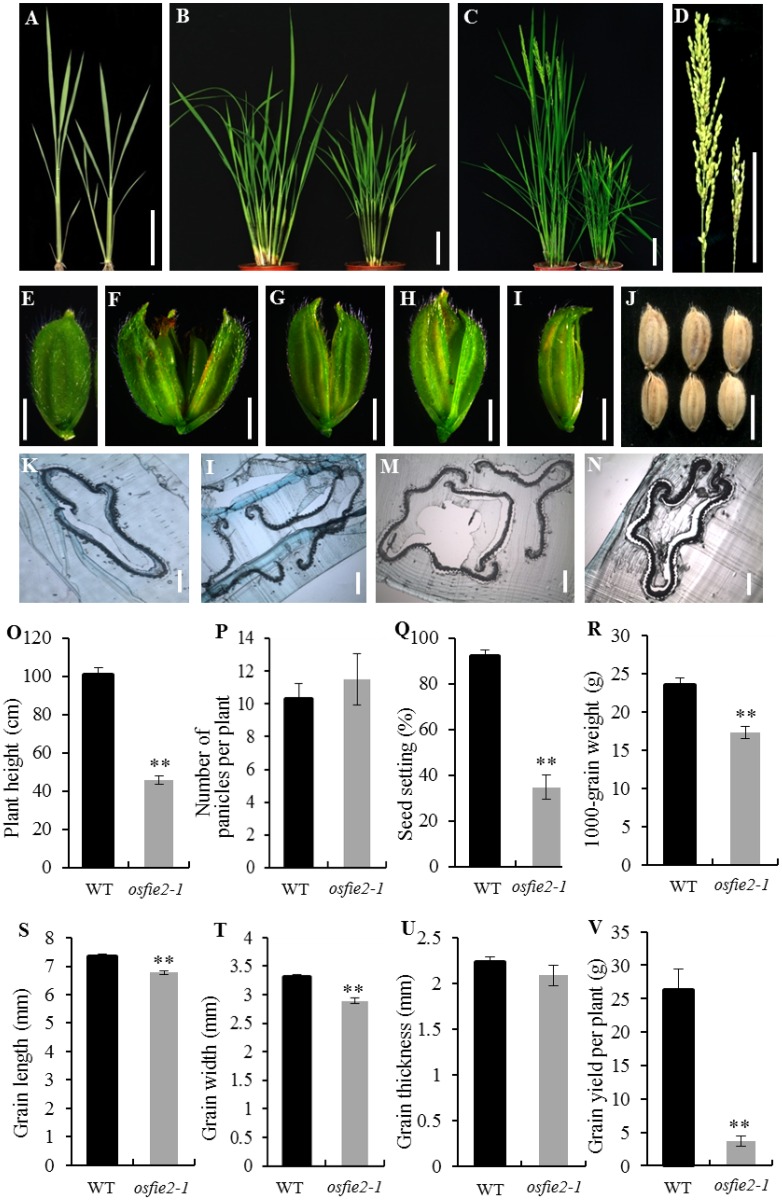
Characterization of the *osfie2-1* mutant. A, The phenotype of the wild-type (*left*) and *osfie2-1* (*right*) during the seedling stage. B, Phenotype of wild-type and *osfie2-1* during the tillering stage. C, Phenotype during the reproductive stage. D, Panicles of wild-type (left) and *osfie2-1* (right). E, Spikelet of wild-type. F-I, Aberrant spikelet of *osfie2-1*. J, The grain phenotype of the wild-type (up) and *osfie2-1* (down). K-N, semi-thin section of *osfie2-1* and wild-type hulls. O-V, Quantification of the phenotypic analysis of wild-type and *osfie2-1*. Data are given as mean ± SD. Student’s t-test was used to generate the P values; * and ** indicate P<0.05 and P<0.01, respectively. Scale bars: 5 cm (A, B); 10 cm (C, D); 0.25 cm (E-I); 0.5 cm (J); 0.05 cm (K-N).

The phenotype of the *osfie2-1* mutant showed no obvious differences before the tillering stage (Fig 1A and 1B). *osfie2-1* presented a markedly dwarf stem, reduced panicle length, narrow leaf, decreased number and size of grains, and suffered from seed sterility problem at maturity compared to wild-type (Fig 1C, 1D, 1J and 1Q; S1A Fig). The stem diameter, leaf length and leaf width were distinctly reduced in *osfie2-1* compared to wild-type, resulting in an overall shorter plant with a final plant height of only about half of that of wild-type (Fig 1O). The decreased grain size of *osfie2-1* was due to the reduction in grain length and grain width, which resulted in significantly lower 1000-grain weight than wild-type (Fig 1R–1T). Taken together, these abnormalities in *osfie2-1* were responsible for a considerably low grain yield per plant, although the number of panicles per plant in *osfie2-1* was not significantly different from wild-type (Fig 1P and 1V). Most of the *osfie2-1* florets were normal however some florets presented abnormal floral morphology owing to the defective development of their palea and lemma. The set of developmental defects in mutant flowers, included elongated lodicules and altered numbers of stamens and pistils, an opened palea or lemma, formation of extra palea or lemma, and sometimes lemma malformation (Fig 1E–1I). Inspection of a hull section from *osfie2-1* and wild-type showed details of these change (Fig 1K–1N). In addition, the heading stage of *osfie2-1* was noted one week earlier than wild-type (data are not shown). The pollen was normal in *osfie2-1* compared to wild-type (S2 Fig).

### Histological analysis of the internode and spikelet hull of *osfie2-1*

**Fig 2:**
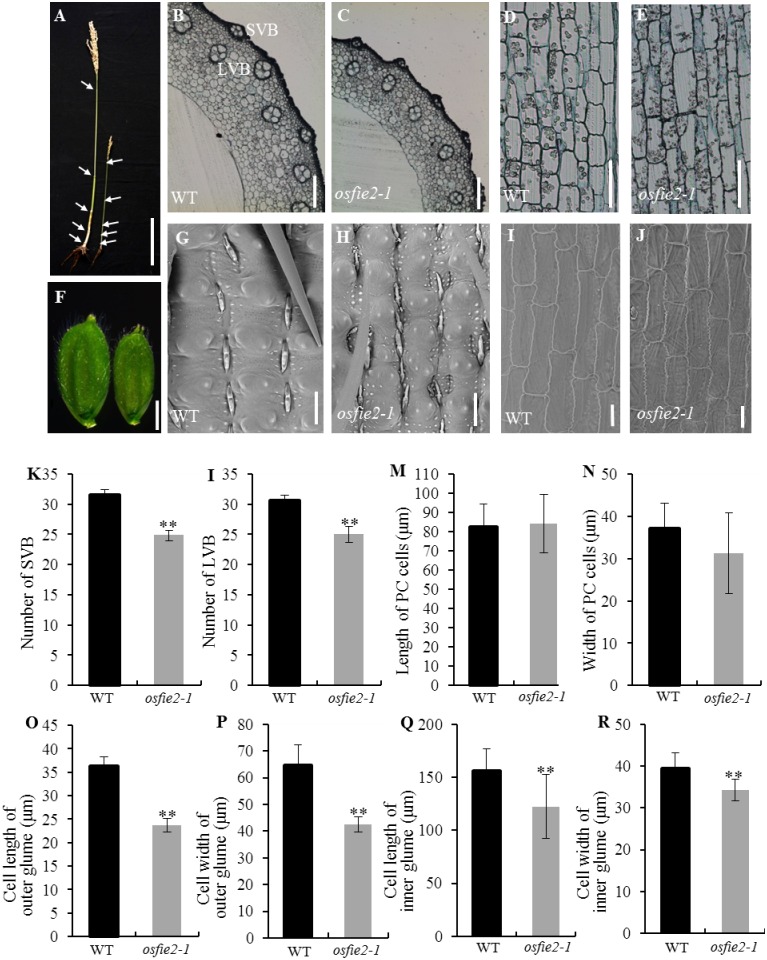
Histological characterization of the stem and spikelet hulls of the *osfie2-1* mutant. A, Comparison of main culms of wild-type and *osfie2-1*, arrows indicate the positions of nodes. B-E, Transverse sections and longitudinal sections of the second internode of the wild-type and *osfie2-1*; SVB, small vascular bundle; LVB, large vascular bundle. F, The spikelet hulls phenotypes of wild-type and *osfie2-1*. G-J, Scanning electron microscope analysis of the outer and inner epidermal cells of spikelet hulls in wild-type and *osfie2-1*. K and L, Number of SVB and LVB calculated from transverse sections of the second internode, (n = 10). M and N, The parenchyma cell (PC) length and width, (n = 12). O-R, Cell length and cell width in outer and inner spikelet hulls, (n = 12). Data are given as mean ± SD. Student’s t-test was used to generate the P values; * and ** indicate P<0.05 and P<0.01, respectively. Scale bars: 15 cm (A); 0.05 mm (B-E); 2 mm (F); 40 μm (G-J).

Compared to wild-type, the length of the panicles and internodes of *osfie2-1* was significantly reduced (Fig 2A and S1B and S1C Fig). Cytology analysis revealed no obvious difference in longitudinal cell size between wild-type and *osfie2-1* (Fig 2D, 2E, 2M and 2N), indicating that the dwarfism of *osfie2-1* could be attributed to the decrease cell number. Moreover, the number of large vascular bundles (LVB) and small vascular bundles (SVB) also decreased in *osfie2-1* (Fig 2K–2I), and the lateral cell layer of *osfie2-1* was markedly thinner than in wild-type, while no difference was observed in their lateral cell number (Fig 2B and 2C). These observations indicated that the thinner culm in *osfie2-1* was due to a reduced VB number and cell layer. The spikelet hulls of *osfie2-1* were much smaller than those of wild-type (Fig 2F). The cell length and width in outer and inner spikelet hulls of *osfie2-1* were significantly reduced in both cell size and cell number (Fig 2G–2J and 2O–2R).

### Genetic analysis and map-based cloning of *osfie2-1*

**Fig 3:**
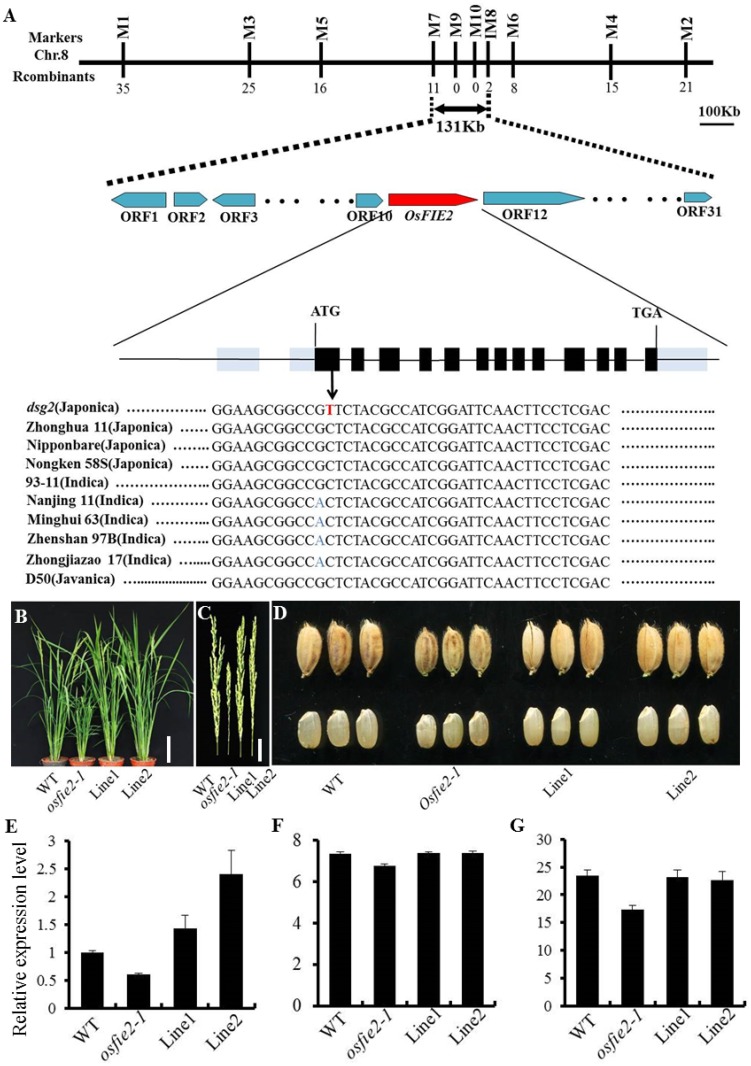
Map-based cloning of *osfie2-1*. A, The *OsFIE2* locus was roughly mapped on the short arm of chromosome 8 between the makers M1 and M2. Fine mapping the *OsFIE2* locus was then restricted to a 131 kb interval between makers M7 and M8. Thirty one candidates genes are illustrated in this region. The number of recombinations are shown under the maker position. A single base change from C to T was found inside the first exon between wild-type (ZH11) and the *osfie2-1* mutant. Eight other varieties all have the wild-type allele. B-D Morphology of wild-type, *osfie2-1* and *OsFIE2* overexperssion transgenic lines, their panicles and grains. E, Relative expression level of *OsFIE2* in wild-type, *osfie2-1* and *OsFIE2* overexperssion transgenic lines. F and G, Grain length and 1000-grain weight in wild type, *osfie2-1* and *OsFIE2* overexperssion transgenic lines. Data are given as mean ± SD. Scale bars: 25 cm (B); 3.5 cm (C); 2.5 mm (D).

The F_1_ plants derived from the reciprocal cross between the *osfie2-1* and its wild-type, between *osfie2-1* and cv. Dular all showed normal phenotype, resembled the wild-type (S3 Fig). And the trait segregated ratio of 3:1 for the normal to mutant plants in each F_2_ population (S1 Table), showing that the mutant trait of *osfie2-1* is inherited as a monogenic recessive nuclear gene. The F_2_ populations derived from the cross between *osfie2-1* and Dular was subsequently used for fine gene mapping. The *osfie2-1* gene was preliminarily located on the short arm of chromosome 8, between the markers M1 and M2. The advanced mapping localized *osfie2-1* at an interval of 131-kb region between the markers M7 and M8, where it co-segregated with the markers M9 and M10. Within this region, 31 open reading frames (ORFs) were predicted, of which ORF11 (Os08g0137100, *OsFIE2*) and ORF14 (Os08g0137250, *OsFIE1*) have been related to plant height [47, 48]. Sequencing analysis showed that the sequence of *OsFIE1* no difference between wild type and mutant, while sequencing comparison of ORF11 between *osfie2-1* and wild-type as well as with other eight varieties showed a single nucleotide change (C to T) in its first exon, resulting in a conversion of Leu (wild-type) into Phe (in *osfie2-1*) at the 40th amino acid (Fig 3A). The expression level of *OsFIE2* was also obviously down regulated in *osfie2-1* (S5 Fig). Thus, we suggest that *OsFIE2* is the candidate gene for dwarf and small grain mutant *osfie2-1*. To confirm this, *OsFIE2* was overexpressed in the *osfie2-1* mutant. The resultant T_0_ transgenic lines did not show any of the mutant phenotypes, including plant height, panicle length and grain size (Fig 3B–3G and S4 Fig), suggesting that *OsFIE2* rescued the mutation.

### Sub-cellular localization and expression profile of *OsFIE2*

**Fig 4:**
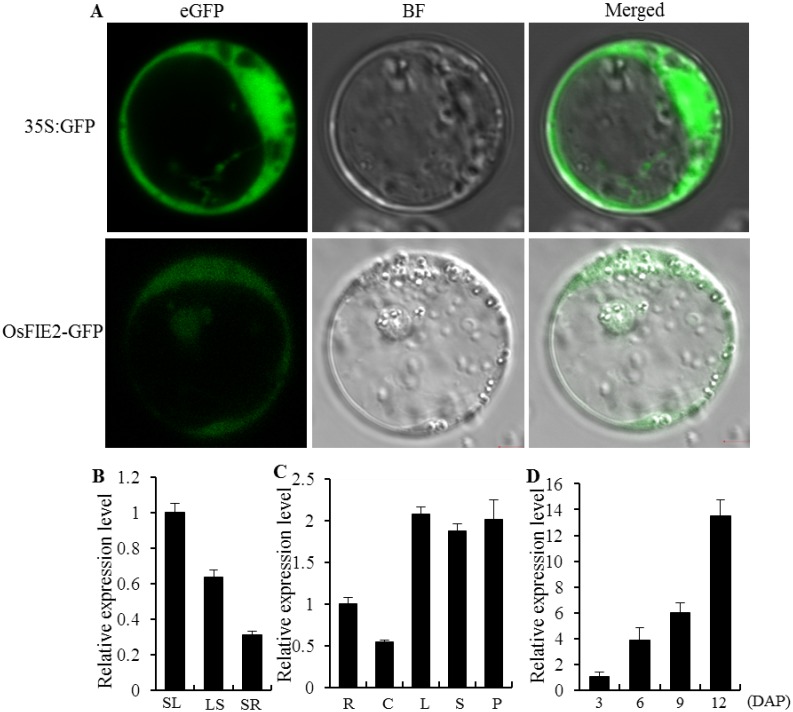
Subcellular localization and expression pattern of *OsFIE2*. A, Subcellular localization of OsFIE2-GFP fusion protein in rice protoplasts. Confocal scanning images show localization in the nucleus and cytoplasm. 35S:GFP was used as a positive control. eGFP, enhanced green fluorescent protein. BF, bright-filed image. B, Expression pattern of *OsFIE2* at the seedling stage. SL, seedling leaves; LS, leaf sheath; SR, seedling roots. C, Expression pattern of *OsFIE2* at the heading stage. R, roots; C, culms; L, leaves; S, leaf sheaths; P, Panicles. D, Expression pattern of *OsFIE2* in the endosperm at different stages (3, 6, 9, 12 days after pollination, DAP). Data are given as mean ± SD of three biological replicates.

When the OsFIE2:GFP fusion protein was transformed into rice protoplasts, the green fluorescence clearly appeared in both the nucleus and cytoplasm (Fig 4A and S6 Fig). We also evaluated the expression pattern of *OsFIE2* by qRT-PCR. The ubiquitous expression of *OsFIE2* was found in all the plant organs examined, including leaves, leaf sheaths, culms, roots, and panicles throughout vegetative and reproductive stages (Fig 4B and 4C). After pollination, the expression level of *OsFIE2* gradually increased in the developing endosperm (Fig 4D).

### OsFIE2 physically interacts with OsiEZ1

**Fig 5:**
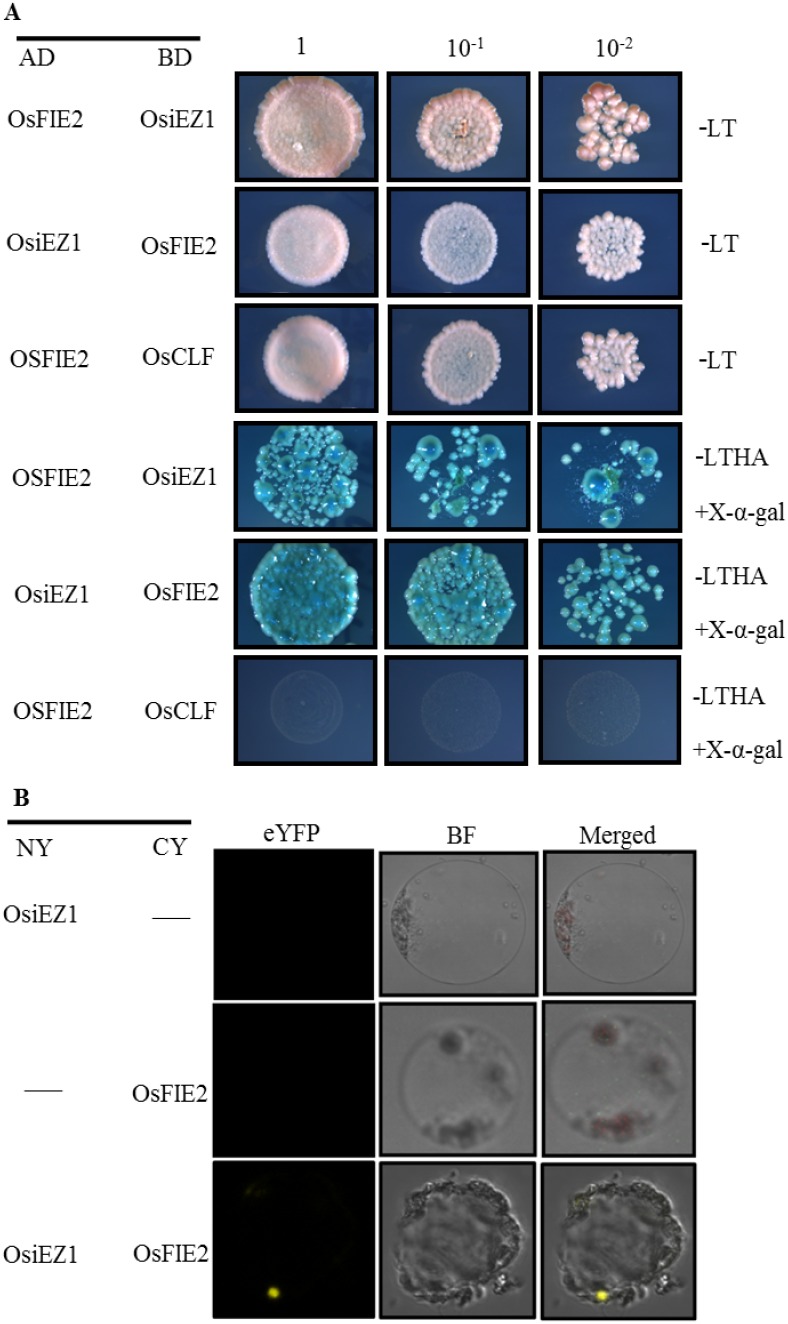
Interaction of OsFIE2 and OsiEZ1. A, Yeast two-hybrid assay showing OsFIE2 interacting with OsiEZ1. The co-transformations with prey and bait were examined on the control media–LT (SD-Trp/-Leu) and the interactions between bait and prey were performed on selective media–LTHA (SD-His/-Trp/-Leu/-Ade/-His) plus X-α-gal. AD, activating medium; BD, binding domain; SD, synthetic dropout. B, Bimolecular fluorescence complementation analysis showing OsFIE2 interacting with OsiEZ1. eYFP, enhanced yellow fluorescent protein. BF, bright-filed image. NY and CY indicate the N terminus and C terminus of eYFP, respectively.

In *Arabidopsis thaliana*, FIE and E(Z) form the PRC2 complex core. E(Z) functions as a histone methylation enzyme. OsiEZ1 and OsCLF are two rice homologues for E(Z) [46]. To determine whether OsFIE2 interacts with either OsiEZ1 or OsCLF, we conducted protein-protein interaction assays. A yeast two-hybrid (Y2H) assay revealed that OsFIE2 physically interacts with OsiEZ1 but not with OsCLF (Fig 5A). This result was further confirmed in vivo via a bimolecular fluorescence complementation (BiFC) experiment using the N and C termini of YFP to reconstitute a functional fluorescent protein (Fig 5B).

### The *osfie2-1* mutation compromised the expression of genes involved in various metabolic pathways

**Fig 6:**
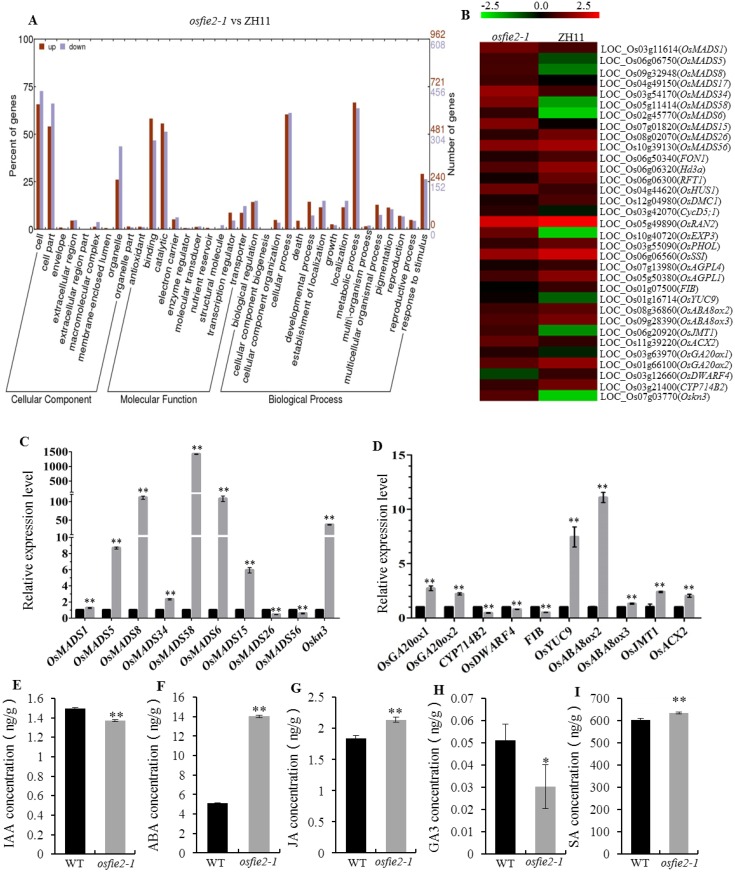
RNA-seq-based and qPCR analysis of differentially expressed genes. A, GO enrichment analysis of differentially expressed genes (DEGs). B, Heatmap showing the expression levels of DEGs in *osfie2-1* according to RNA-seq. C, Expression level of homeobox *OsMADS-BOX* genes and *Oskn3* in wild-type and *osfie2-1*. D, Expression level of phytohormone-related genes. E-I, Phytohormones content in wild-type and *osfie2-1*. Data are given as mean ± SD of three biological replicates. Student’s t-test was used to generate the P values; * and ** indicate P<0.05 and P<0.01, respectively.

An RNA-sequencing-based transcriptome analysis was performed between wild-type and *osfie2-1* to further clarify the function of *OsFIE2*. A total of 1,747 genes were differentially expressed, and among them, 1,078 genes were up-regulated in *oosfie2-1*, while the remaining 669 were down-regulated. The functional information of these genes was carried out by using Gene Ontology (GO) analysis (http://www.geneontology.org/). Considering their molecular function annotation, most of the differentially expressed genes were involved in catalytic and binding activity. At the same time, the majority of these genes were associated with many biological processes, including metabolic process, cellular process, and response to stimulus (Fig 6A and S6 Table).

Further scrutiny of the differentially expressed genes revealed that the expression of a set of genes with known function were up- or down-regulated. These were homeobox *MADS-box* genes involved in floral development [51], *FON1* that regulates floral organ number [52], *Hd3a* and *RFT1* which are related to heading stage [53], *OsHUS1* and *OsDMC1* associated with meiosis [54, 55], *CycD5;1*, *OsRAN2* and *OsEXP3* that affect cell cycle and cell expansion [56, 57], *OsSSI*, *OsAGPL1* and *OsAGPL4* implicated in starch synthesis in the endosperm [48], the homeobox gene *Oskn3* related to internode architecture, and *FIB*, *OsYUC9*, *OsABA8ox2*, *OsABA8ox3*, *OsGA20ox1*, *OsGA20ox2*, *OsDWARF4*, *CYP714B2*, *OsJMT1* and *OsACX2* which are associated with the synthesis or signaling of the phytohormones auxin (IAA), abscise acid (ABA), gibberellic acid (GA) and jasmonic acid (JA) [23, 58–66] (Fig 6B). The transcription profile of some of these genes was confirmed by QRT-PCR (Fig 6C and 6D). Analysis of phytohormone contents showed that IAA, ABA, GA, JA and SA all accumulated in the *osfie2-1* mutant (Fig 6E–6I). The expression level of those genes associated with various metabolic pathways up or down-regulated may lead to the mutant occur abnormal phenotypes and the abnormal accumulation of phytohormones in *osfie2-1*.

## Discussion

Polycomb proteins regulate extensive developmental processes in plants. In this study, we identify a dwarf and small grain mutant *osfie2-1*, and found that a fertilization independent endosperm protein OsFIE2 was responsible for the abnormal phenotype of *osfie2-1* (Fig 3). We found that *osfie2-1* mutant produced pleiotropic and effects on agronomic traits, exhibited markedly shorter and thinner culms, characterized by smaller panicles and gain size when compared to the wild-type (Fig 1A–1D and 1J), and also displayed abnormal floral organs (Fig 1E–1I). Finally, *osfie2-1* also presented a considerably low grain yield per plant (Fig 1V). In previous research, the characteristics of *OsFIE2* gene knock down line by RNAi technique have been already reported. The RNAi lines also shown pleiotropic phenotypes in vegetative and reproductive organ generation, such as dwarf, abnormal enlarge lemma and small gain. In *osfie2-1* mutant, only *OsFIE2* was down regulated (S5 Fig), whereas, expression levels of both *OsFIE1* and *OsFIE2* were reduced in *OsFIE2* RNAi lines [33]. So, *osfie2-1* mutant is more helpful in the study of the function of OsFIE2 by eliminated the interference of the *OsFIE1*. Histological analysis found that that the longitudinal cell number in internodes decreased in *osfie2-1*, and the lateral cell layer of the internodes was markedly thinner than in wild-type, whereas there was no difference in the number of lateral cells between wild-type and *osfie2-1*. In addition, the number of LVB and SVB decreased in *osfie2-1*, as well as the cell number and size of spikelet hulls (Fig 2). All these observations may explain the dwarfism of these mutants and the smaller size of their grains.

In *Arabidopsis*, FIE protein regulates endosperm and embryo development and represses flowering during embryo and seedling development [67]. Moreover, FIE has also been shown to be essential for controlling shoot and leaf development [41]. Rice orthologs of FIE, OsFIE1 and OsFIE2, have also been identified. A recent study showed that mutation in *OsFIE1* causes a dwarf stature and various floral defects [47]. It was established that *OsFIE1* is regulated by DNA methylation and histone H3K9me2 and is involved in H3K27me3-mediated gene repression [47]. Another research group associated the function of *OsFIE2* to endosperm development [48], however the mechanism of how *OsFIE2* controls grain filling and yield in was not fully described nor was any other effect on plant growth. In our study, we distinguished and described multiple atypical morphologies in the a mutant of *OsFIE2*, *osfie2-1*, such as dwarfism, small grain size, narrow leaf and abnormal floral organs. *OsFIE2* expresses in all tissues, which differs from *OsFIE1* which is expressed only in the endosperm [46, 47]. We also detect OsFIE2 localized in both the nucleus and cytoplasm (Fig 4A and S6 Fig), which agrees with a previous report [33]. In *Arabidopsis*, as two-core component of PRC2-like complexes, FIE and E(Z) proteins physically interact [41, 68]. In our case, the physical interaction between OsFIE2 and OsiEZ1 (the rice E(Z) ortholog) was also demonstrated (Fig 5). Thus, we postulate that OsFIE2 may function through PRC2-like complexes to control growth and development by posttranslational modifications similarly to what was described in *Arabidopsis*.

Down-regulation of the *Arabidopsis FIE* gene produced dramatic morphological aberrations resulting from de-repression of *KNOTTED*-like homeobox and *MADS-box* genes [41]. Significantly reduced expression levels of *OsFIE2* lead to pleiotropic aberrant phenotypes in *OsFIE2* RNAi lines and ectopic expression of some key development regulators, such as the *MADS-box* (*OsMADS3*) and *KNOX* (*Oskn3*) genes [33]. Morphological changes were also evident in *osfie2-1*, thus we performed an RNA-sequencing-based transcriptome analysis and found that there are many genes up or down-regulated in *osfie2-1* (Fig 6). Among them, the expression levels of homeobox genes (*MADS-box* and *Oskn3*) also changed significantly in *osfie2-1* suggesting a probable cause for the abnormalities in floral and seed morphology as well as plant height. Moreover, the different expression levels of cell division/expansion-related genes in *osfie2-1* compared to wild-type may explain the reduction in the cell number and cell size in the internodes and spikelet hulls in the *osfie2-1* mutant. In addition, there were several hormone-related genes differently expressed between the wild-type and *osfie2-1*, which may lead to a different content of ABA, IAA, GA_3_, SA, and JA in *osfie2-1* compared to wild-type. In fact, it has been established that PcG proteins can target genes involved in the biosynthesis, transport, perception and signaling of phytohormones in *Arabidopsis*, specially those implicated in promoting growth [69, 70]. In this regard, an *Arabidopsis* PcG gene, *CLF*, was shown to control both cell division and elongation during leaf expansion [71]. Because of OsFIE2 function through PRC2-like complexes to control growth and development by posttranslational modifications [48], some the up-regulated genes may be directly controlled by OsFIE2 via epigenetic repressive marks, however most differential expression genes may be indirectly regulated by OsFIE2. Of course all of these need further experiments to prove. In summary, our data demonstrates that *OsFIE2* plays a role in determining plant height and grain yield in rice and support the idea that PcG genes such as *OsFIE2*, *OsFIE1* and *OsiEZ1* are essential for growth and development.

## Supporting Information

S1 Fig Analysis of leaf and internodes in *osfie2-1*. A, Comparison of leaf between wild-type (*left*) and *osfie2-1* (*right*). B, Comparison of internode length of the main culm between wild-type (*left*) and *osfie2-1* (*right*), I-V, top-one to top-five internodes, P, Panicle. C, Internode lengths of the wild-type and *osfie2-1*. The results are mean ± SD of 12 independent assays.
(TIF)

S2 Fig KI-I_2_ staining of *osfie2-1* pollen. A, wild-type. B, *osfie2-1*.
(TIF)

S3 Fig Genetic analysis of the *osfie2-1*. A, Comparison of wild-type, *osfie2-1* and heterozygous (F_1_) plants (WT×*osfie2-1*) at the heading stage. B, Comparison of wild-type, *dsg2* and heterozygous (F_1_) panicles (WT×*osfie2-1*). C, Comparison of wild-type, *osfie2-1* and heterozygous (F_1_) plants (*osfie2-1*×WT) at the heading stage. D, Comparison of wild-type, *dsg2* and heterozygous (F_1_) panicles (*osfie2-1*×WT). E, Sequencing peak pattern of the heterozygous (F_1_) plants (WT×*osfie2-1*). Arrows indicate heterozygous loci (C/T). F, Sequencing peak pattern of the heterozygous (F_1_) plants (*osfie2-1*×WT). Arrows indicate heterozygous loci (C/T).
(TIF)

S4 Fig Transverse and longitudinal sections of WT and *OsFIE2* overexpression transgenic lines second internodes. Scale bars, 0.05 mm (A-D).
(TIF)

S5 Fig The expression of *OsFIE2* at heading stage. Data are given as mean ± SD. Student’s t-test was used to generate the P values; * and ** indicate P<0.05 and P<0.01, respectively.
(TIF)

S6 Fig The Subcellular Localization of *OsFIE2*. (TIF)

S1 Table Segregation of F2 progeny from the heterozygous (F1) plant. (DOCX)

S2 Table Primers used in fine mapping. (DOCX)

S3 Table Sequencing primers. (DOCX)

S4 Table Primers used in vector constructions. (DOCX)

S5 Table Primers used in qPCR. (DOCX)

S6 Table Differential expressed genes in *osfie2-1*. (XLSX)
