# Supplementary figures and images for: Polycomb Protein OsFIE2 Affects Plant Height and Grain Yield in Rice
Source: PLoS One. 2016 Oct 20;11(10):e0164748. doi: 10.1371/journal.pone.0164748 (PMC5072591; doi:10.1371/journal.pone.0164748)

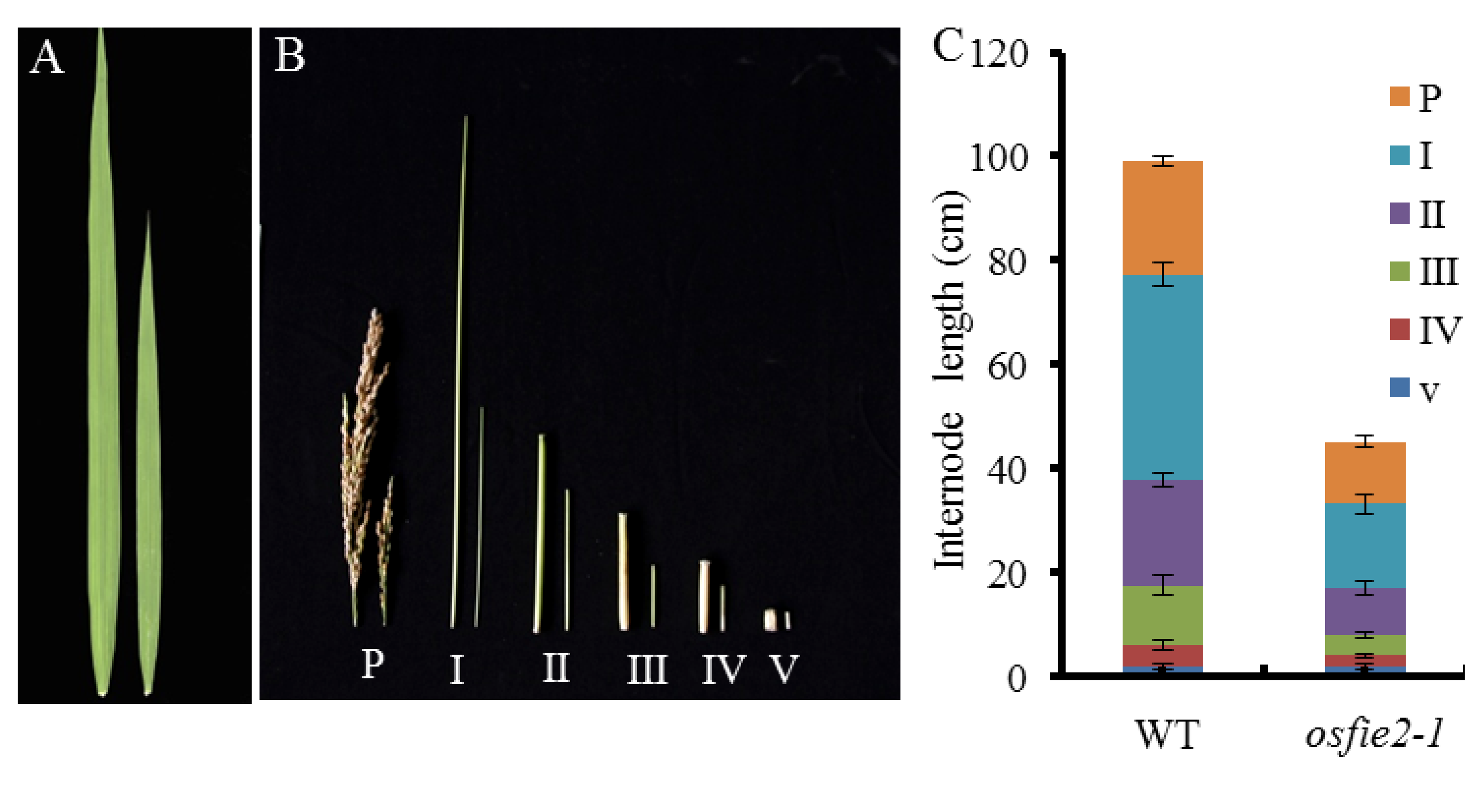

Supplement: S1 Fig — A, Comparison of leaf between wild-type (left) and osfie2-1 (right). B, Comparison of internode length of the main culm between wild-type (left) and osfie2-1 (right), I-V, top-one to top-five internodes, P, Panicle. C, Internode lengths of the wild-type and osfie2-1. The results are mean ± SD of 12 independent assays. (TIF) [file pone.0164748.s001.tif]

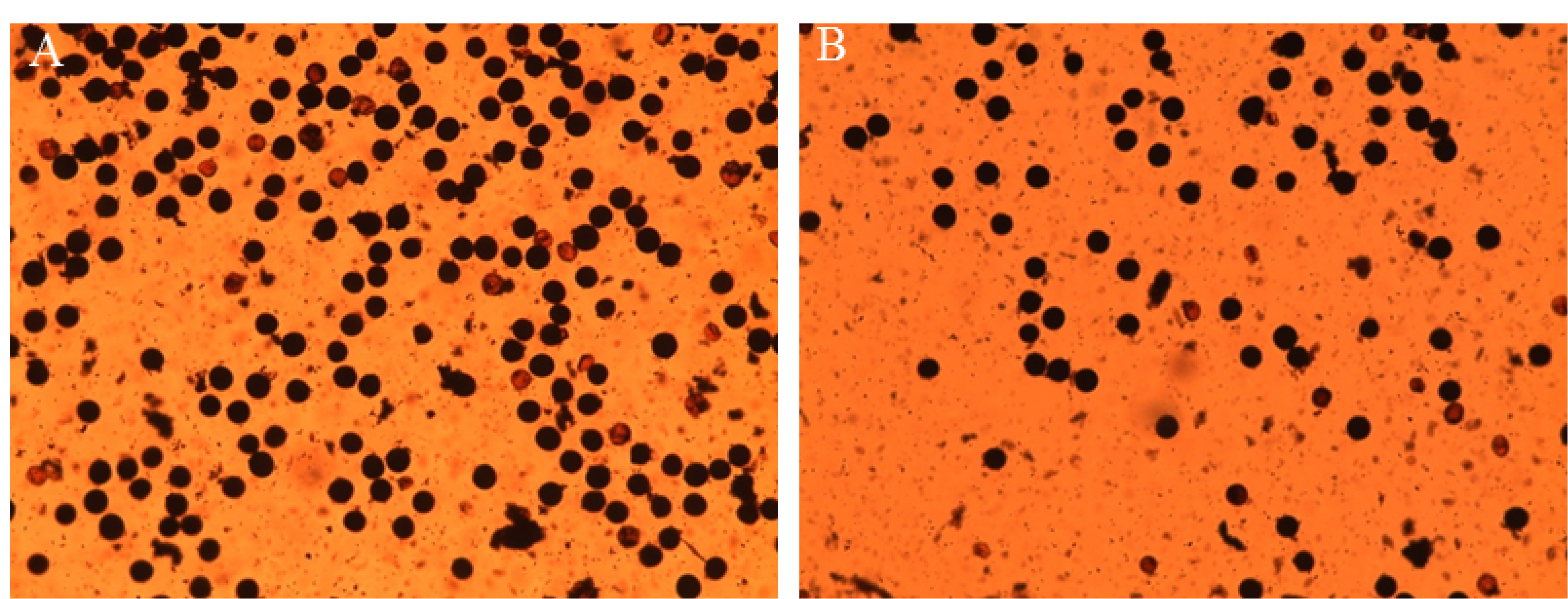

Supplement: S2 Fig — A, wild-type. B, osfie2-1. (TIF) [file pone.0164748.s002.tif]

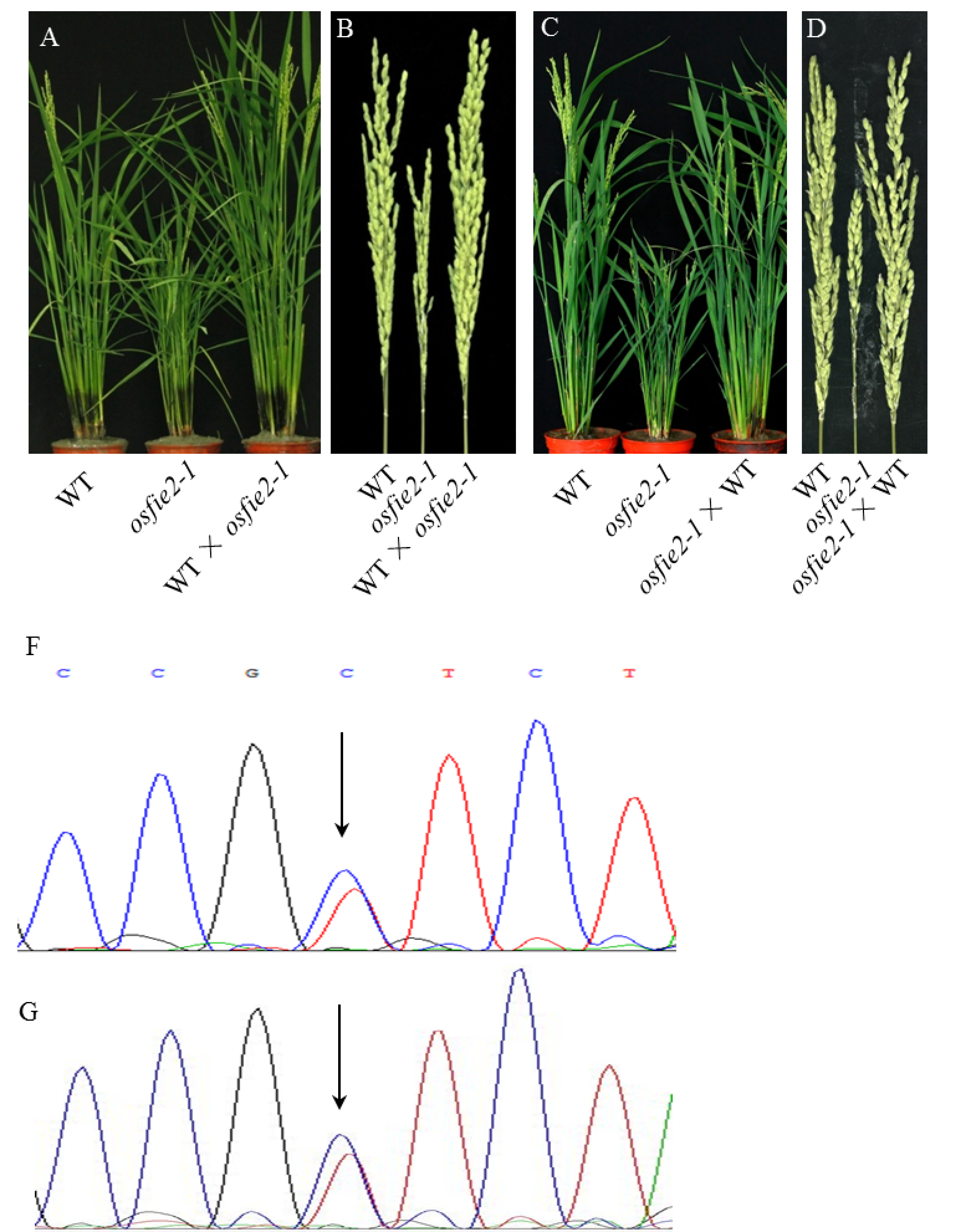

Supplement: S3 Fig — A, Comparison of wild-type, osfie2-1 and heterozygous (F1) plants (WT×osfie2-1) at the heading stage. B, Comparison of wild-type, dsg2 and heterozygous (F1) panicles (WT×osfie2-1). C, Comparison of wild-type, osfie2-1 and heterozygous (F1) plants (osfie2-1×WT) at the heading stage. D, Comparison of wild-type, dsg2 and heterozygous (F1) panicles (osfie2-1×WT). E, Sequencing peak pattern of the heterozygous (F1) plants (WT×osfie2-1). Arrows indicate heterozygous loci (C/T). F, Sequencing peak pattern of the heterozygous (F1) plants (osfie2-1×WT). Arrows indicate heterozygous loci (C/T). (TIF) [file pone.0164748.s003.tif]

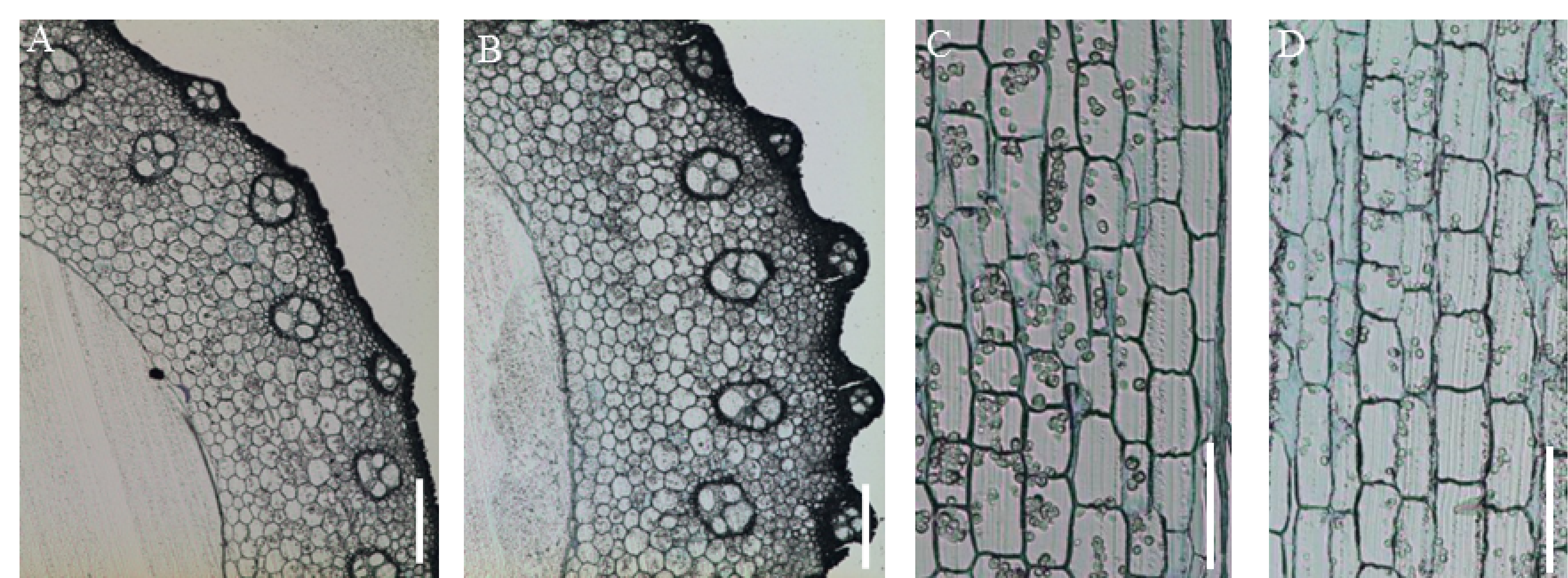

Supplement: S4 Fig — Scale bars, 0.05 mm (A-D). (TIF) [file pone.0164748.s004.tif]

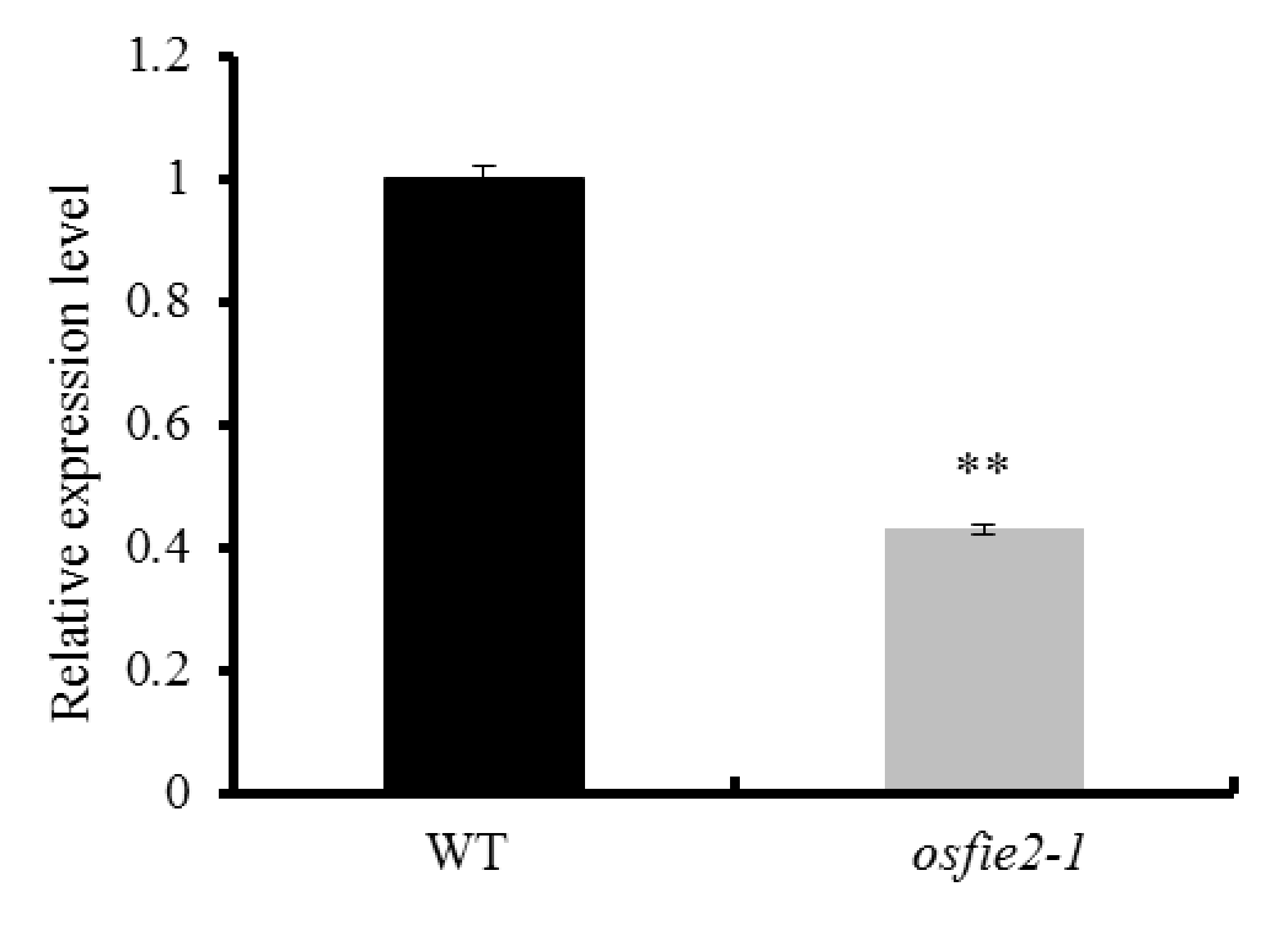

Supplement: S5 Fig — Data are given as mean ± SD. Student’s t-test was used to generate the P values; * and ** indicate P<0.05 and P<0.01, respectively. (TIF) [file pone.0164748.s005.tif]

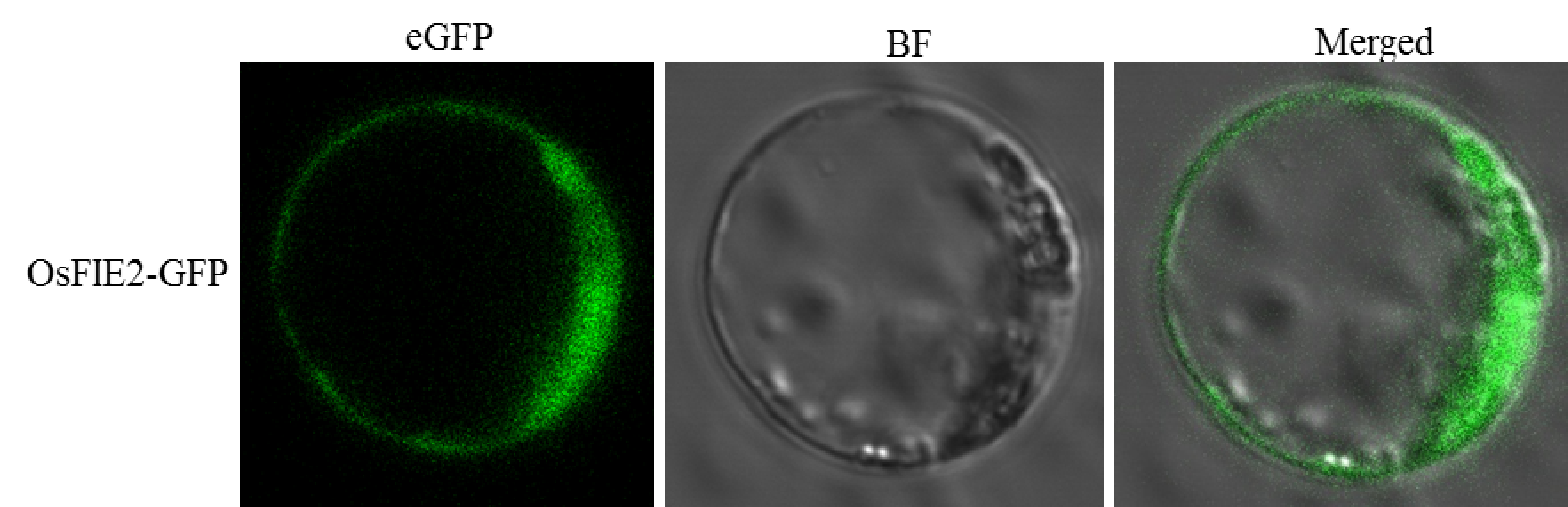

Supplement: S6 Fig — (TIF) [file pone.0164748.s006.tif]
