# Supplementary material for: Polycomb Protein OsFIE2 Affects Plant Height and Grain Yield in Rice
Source: PLoS One. 2016 Oct 20;11(10):e0164748. doi: 10.1371/journal.pone.0164748 (PMC5072591; doi:10.1371/journal.pone.0164748)
Supplement: S1 Table — (DOCX) [file pone.0164748.s007.docx]

**S1 Table. Segregation of F2 progeny from the heterozygous (F1) plant.**

| Population | Total | Normal | Mutant | Expected value | χ^2^ valve |
| --- | --- | --- | --- | --- | --- |
| WT×*osfie2-1* | 168 | 121 | 47 | 3:1 | 0.37 |
| *osfie2-1*×WT | 283 | 201 | 82 | 3:1 | 0.12 |
| *osfie2-1*×Dular | 565 | 441 | 124 | 3:1 | 0.094 |
