# Supplementary material for: Polycomb Protein OsFIE2 Affects Plant Height and Grain Yield in Rice
Source: PLoS One. 2016 Oct 20;11(10):e0164748. doi: 10.1371/journal.pone.0164748 (PMC5072591; doi:10.1371/journal.pone.0164748)
Supplement: S2 Table — (DOCX) [file pone.0164748.s008.docx]

**S2 Table. Primers used in fine mapping.**

| Markers | Primers sense (5’–3’) | Anti-sense (5’–3’) |
| --- | --- | --- |
| M1 | GCCTACTCAGCTTCCTCTCCTTCG | CGCCATTTACGGCAGCAACC |
| M2 | TTTCTCTCCACTTCTTCCTTCTCC | CACCATCGCTACCTTCTCTTCC |
| M3 | CACGTTCACAACTCACTGGG | TTATCTGCCACCTGAGTCCC |
| M4 | CCTTCGACGACCTCAGTTTGG | ATTTAAAGGAACCCGACCGATCC |
| M5 | CATCAGCTCGCATCATCACT | AGCCGCTCCCTTCATCTT |
| M6 | ACCTCGTCTTCCTCCTCCAT | AGCTCGATAGCTAGGTCGCA |
| M7 | CCCACCGATCGAGAATCTAA | AGGATTCGTGCAAGCTGAGT |
| M8 | GGGATGTGGTACATTCTAGT | TAGTGGTATCAGCCTGGTAG |
| M9 | ACGAGCTCTCGATCAGCCTAGC | CACTCCATGGAAGAGGCAAGC |
| M10 | AATTAGCTTGCAGCGGAATCACATGC | GACGGAGGGAGTAGCGATTTGTCC |
