# Supplementary material for: Polycomb Protein OsFIE2 Affects Plant Height and Grain Yield in Rice
Source: PLoS One. 2016 Oct 20;11(10):e0164748. doi: 10.1371/journal.pone.0164748 (PMC5072591; doi:10.1371/journal.pone.0164748)
Supplement: S3 Table — (DOCX) [file pone.0164748.s009.docx]

**S3 Table. Sequencing primers.**

| Markers | Primers sense (5’–3’) | Anti-sense (5’–3’) |
| --- | --- | --- |
| fie2-1 | GGAGTGAGGTGGAATAAGAT | TGACTATGGGTAGTTTGTGG |
| fie2-2 | CCTCTGCCTAGCTCCACCTA | TGCATTGTAACGGATGAAAA |
| fie2-3 | TGAGAACATAATAAAATGCAAG | CGAAGACCTCGTAGTAGTGG |
| fie2-4 | CTTTTGCTCGCCCTACTAAC | AGCCCACTAAAGACAACCATC |
| fie2-5 | ATTACGCAGATTTTGGTTCG | TGCCCTATTTTGTGGTGTTC |
| fie2-6 | TGCTTCAGATAATAGTTGCT | CATCATACTGGGAAATAGAA |
| fie2-7 | GATGAATCTGTTAGGCTGTG | GAATCCTACTGAAGTTTTGC |
| fie2-8 | TGGCATCATACCAATCTTTC | CTTGGAACTTACCGAGCAGT |
| fie2-9 | CAGCACCCTTAATGTTGTGA | TTTTCCAAAAATCTGGCATAGCCGA |
| fie2-10 | CAGCTTTTACTTGCCCACAA | TATAGGGTTTGTTTGGTTTACAGCC |
