# Supplementary material for: Polycomb Protein OsFIE2 Affects Plant Height and Grain Yield in Rice
Source: PLoS One. 2016 Oct 20;11(10):e0164748. doi: 10.1371/journal.pone.0164748 (PMC5072591; doi:10.1371/journal.pone.0164748)
Supplement: S4 Table — (DOCX) [file pone.0164748.s010.docx]

**S4 Table. Primers used in vector constructions.**

| Markers | Primers sense (5’–3’) | Anti-sense (5–3’) |
| --- | --- | --- |
| p1390-OsFIE2 | TTACTTCTGCACTAGGTACCATGGCGAAGCTGGGGCCGGG | GAATTCCCGGGGATCCTCATGCTTTTGGATGGTCCA |
| pAN580-OsFIE2 | GCCCAGATCAACTAGTATGGCGAAGCTGGGGCCGGG | TGCTCACCATGGATCCTGCTTTTGGATGGTCCACTT |
| pGBT9-OsFIE2 | TGTATCGCCGGAATTCATGGCGAAGCTGGGGCCGGG | CGGAATTAGCTTGGCTGCAGTGCTTTTGGATGGTCCACTT |
| pGAD-OsFIE2 | GGAGGCCAGTGAATTCATGGCGAAGCTGGGGCCGGG | CGAGCTCGATGGATCCTGCTTTTGGATGGTCCACTT |
| pGBT9-OsiEZ1 | TGTATCGCCGGAATTCATGGCGTCGTCCTCGTCCAA | CGGAATTAGCTTGGCTGCAGTCTAGCAACTTTGTGCGCTC |
| pGAD-OsiEZ1 | GGAGGCCAGTGAATTCATGGCGTCGTCCTCGTCCAA | CGAGCTCGATGGATCCTCTAGCAACTTTGTGCGCTC |
| pGBT9-OsCLF | TGTATCGCCGGAATTCATGGCTGGCGATTCCCGAAA | CGGAATTAGCTTGGCTGCAGGTGGGCGAGCTTCTTTGCTC |
| pSPYCE-OsFIE2 | CGCCACTAGTGGATCCATGGCGAAGCTGGGGCCGGG | TACCCTCGAGGTCGACTGCTTTTGGATGGTCCACTT |
| pSPYNE-OsiEZ1 | CGCCACTAGTGGATCCATGGCGTCGTCCTCGTCCAA | TACCCTCGAGGTCGACTCTAGCAACTTTGTGCGCTC |
