# Supplementary material for: Polycomb Protein OsFIE2 Affects Plant Height and Grain Yield in Rice
Source: PLoS One. 2016 Oct 20;11(10):e0164748. doi: 10.1371/journal.pone.0164748 (PMC5072591; doi:10.1371/journal.pone.0164748)
Supplement: S5 Table — (DOCX) [file pone.0164748.s011.docx]

**S5 Table. Primers used in qPCR.**

| Markers | Primers sense (5’–3’) | Anti-sense (5’–3’) |
| --- | --- | --- |
| *Ubiquitin* | GCTCCGTGGCGGTATCAT | CGGCAGTTGACAGCCCTAG |
| *OsMADS1* | GTCTTCTCCACCCTCACC | TGTCTGCTGCTTCATTGC |
| *OsMADS5* | GGTCGCCCTCATCATCTT | TCGGGTCCTGCGTAACTGC |
| *OsMADS8* | AAGCAGCCGCAATGAGTA | TATGCCAAGTGTCCCAAG |
| *OsMADS34* | TCGCCCTCGTCCTCTTCT | GCATCTCATCGCTAGTTGGT |
| *OsMADS58* | CGAGAACACGACGAACCG | TGGAGAAGACGACGAGGG |
| *OsMADS6* | GGAACTTCGCAGAAAGGA | GGCTCTGTAGTTGCTGGTG |
| *OsMADS15* | AGCCCAGACAAGCTCCTC | CGCATCATTTCTCTCGCCCATCATC |
| *OsMADS26* | CTCTACGACCTCGCCACCA | GAGCACCATTGCCTCCTG |
| *OsMADS56* | TGAAGCGGATTGAGAACC | GGGAGAAGACGATGAGGG |
| *OSH15* | CAACCAGAGGAAACGGCACT | CGAACCGAGGCGGTACATT |
| *OsGA20ox1* | GAGGAGGGCGTGGGTGAGTA | CCATCAGCTCCAGCGACAGG |
| *OsGA20ox2* | GCCGACTACTTCTCCAGCACCC | CGTCAGCGACAGCTCCTTCATC |
| *CYP714B2* | GCTGCCTGGTGTCTAATG | TGGGTACAAACGCAAAGT |
| *OsDWARF4* | GGAAGAGCGGGTTGAGAAG | TCAGCAAGAGGTCCAGGATT |
| *FIB* | TCCGGCTTCTGCAACTTCA | ACAATCCTCCACATCCTCCCTA |
| *OsYUC9* | CGGGCGAGAACGACGAGA | GCACGGACTTCCCCTTGAAC |
| *OsABA8ox2* | TCCAGCTCTACTCCCAGGACC | CTGCGACACCAGCACGAA |
| *OsABA8ox3* | CGAACACCTTCATGCCATTT | CCACCTGTAGCCAGTGACCAG |
| *OsACX2* | GAGGATGTTATGGCTGATGT | GATTCTCGGCATACGCTA |
